# Counterfactual learning in enhancing resilience in autonomous agent systems

**DOI:** 10.3389/frai.2023.1212336

**Published:** 2023-07-28

**Authors:** Dilini Samarasinghe

**Affiliations:** School of Systems and Computing, University of New South Wales, Canberra, ACT, Australia

**Keywords:** autonomous agent systems, multi-agent system (MAS), resilience, counterfactual learning, machine learning, explainability, explainable agents, robustness

## Abstract

Resilience in autonomous agent systems is about having the capacity to anticipate, respond to, adapt to, and recover from adverse and dynamic conditions in complex environments. It is associated with the intelligence possessed by the agents to preserve the functionality or to minimize the impact on functionality through a transformation, reconfiguration, or expansion performed across the system. Enhancing the resilience of systems could pave way toward higher autonomy allowing them to tackle intricate dynamic problems. The state-of-the-art systems have mostly focussed on improving the redundancy of the system, adopting decentralized control architectures, and utilizing distributed sensing capabilities. While machine learning approaches for efficient distribution and allocation of skills and tasks have enhanced the potential of these systems, they are still limited when presented with dynamic environments. To move beyond the current limitations, this paper advocates incorporating counterfactual learning models for agents to enable them with the ability to predict possible future conditions and adjust their behavior. Counterfactual learning is a topic that has recently been gaining attention as a model-agnostic and post-hoc technique to improve explainability in machine learning models. Using counterfactual causality can also help gain insights into unforeseen circumstances and make inferences about the probability of desired outcomes. We propose that this can be used in agent systems as a means to guide and prepare them to cope with unanticipated environmental conditions. This supplementary support for adaptation can enable the design of more intelligent and complex autonomous agent systems to address the multifaceted characteristics of real-world problem domains.

## 1. Introduction

In the fast-paced society of today, the application demands for collective intelligence are increasing. Applications of autonomous systems are often found in fields that require distributed sensing and action. These include both engineering-related disciplines that are interested in autonomous self-organizing artifacts and science-related disciplines that are interested in understanding the behaviors and evolution of the natural world. The benefits in safety, productivity, and in saving money and time has induced intelligent systems use to be more widespread across a range of domains (Singh et al., 2013). Research related to autonomous aerial (Kulkarni et al., 2022), aquatic (Bu et al., 2022), and ground (Chakraborty et al., 2022) agent systems has demonstrated interest in domains such as search and rescue (Huamanchahua et al., 2022), exploration (Azpúrua et al., 2023), planning (Raja and Pugazhenthi, 2012), and surveillance (Filipović et al., 2023). In the sense of industrial applications, autonomous intelligent systems are sought after in fields such as healthcare (Kyrarini et al., 2021), manufacturing (Shneier and Bostelman, 2015), retail (Bogue, 2019), military (Voth, 2004), and mining (Nanadrekar et al., 2022).

Advances in these domains cannot be achieved without automation that can facilitate intelligent capabilities primarily related to learning, adaptation, and evolution. Learning in an agent system can be viewed from two perspectives as: passive learning and active learning (Taylor et al., 2021). When prior-knowledge is abundant and the environment is not dynamic, passive learning is useful to construct the parametric representations of relationships within the model. However, intelligent systems that can make decisions to best realize goals in dynamic environments require individual agents that follow an active learning policy where they can understand and exploit the interactions they encounter. Active learning sees a wide range of applications particularly in terms of prioritized decision-making; object recognition, detection, and classification; and inspection related tasks. It is also not sufficient for agent systems to be *functionally adequate* to address the complex problems in the current application domains. They need to be adaptive, thus, have the ability to self organize (Bernon et al., 2005). The components or the individual agents in the system should be endorsed with the capability to rearrange their behaviors and relations with each other locally without the requirement of manual intervention of a programmer. Adaptive characteristics in an agent system are specifically appreciated for requirements such as maintaining intrinsic safety and learning and accomplishing new tasks. The capacity to evolve its characteristics and functionality in an agent system is closely related to the requirements of learning and adaptation as well. While autonomous agent systems have progressed a long way in terms of accuracy and speed of completing monotonous tasks compared to their human counterparts, they have not yet reached the level of cognition possessed by humans that could ideally lead to modifications and advancements in the system's design based on an evolutionary process. Autonomous evolution in such systems would facilitate long term existence of agent ecosystems that can survive in diverse environmental niches and specialize in different tasks without direct oversight from human designers (Lan et al., 2021).

These expectations of agent systems reveal that there exists a requirement for them to have the capacity to anticipate, respond to, adapt to, and recover from adverse and dynamic conditions in complex environments. This can be attributed to the resilience of these systems which facilitates incorporating agile policies that allow adaptation to newly perceived conditions and overcome disturbances that were not modeled. In a resilient system, the agents should possess the intelligence to apply a transformation, reconfiguration, or an expansion across the system to preserve their functionality, change their functionality, or to minimize the impact on functionality (Prorok et al., 2021).

The early work related to adapting and overcoming unexpected encounters within agent systems have primarily focused on parametric uncertainty (Luders et al., 2010) and fault tolerance (Parker, 1998). Although these adaptive control methods can alter process dynamics to suit the conditions faced, they are restricted by design assumptions and conditions (Barber et al., 2000; Åström and Wittenmark, 2013). While they accommodate uncertainty and risk, these models are mostly concerned with the sensitivity of the system output to parametric changes and bounded disturbances that are within expected design conditions. In contrast, resilient agent systems are more relaxed on such assumptions and investigate the capability of adapting to and withstanding disturbances and adverse conditions which have not been modeled (Prorok et al., 2021).

In this perspective, the dynamic and adverse conditions and environmental states should be addressed through agile approaches that can facilitate system-wide transformations. This paper emphasizes the need for resilient agent systems to anticipate unmodeled disturbances and leverage emerging behaviors to retain desired performance levels. Resilience can be discussed in two contexts under morphological and behavioral aspects. The focus here is on achieving resilience through improvements to the behavioral aspects of the agents rather than improving the morphological aspects such as self-reconfigurability of hardware components.

Toward this end, Section 2 identifies the state-of-the-art in resilient autonomous agent systems and approaches to improve resilience which are limited in their capacity to anticipate adverse conditions. Means of addressing this limitation through counterfactual learning is presented in Section 3. This concept is gaining attention in the research community as a mode of explaining and making inferences on past, present, and future relations within AI systems. In this paper, the application of counterfactual learning is extended toward maintaining resilience of agent systems. Ways of using counterfactual causality to help gain insights into unforeseen circumstances and make inferences about the probability of an outcome are presented in Section 3. This section identifies counterfactual learning as an approach to guide and prepare them to cope with unanticipated environmental conditions. Finally, Section 5 concludes the paper with a potential way forward in implementing the proposed approach and a discussion of the current perspectives and limitations of the approach.

## 2. Resilience in autonomous agent systems

As discussed in Section 1, collective intelligence is a highly demanding attribute for agent systems, but, is equally challenging to automate. Specifically, automating the strategic planning process for identifying conditions for purposeful decision making requires the agents to follow an active and adaptive learning architecture (Samarasinghe et al., 2022a) It is associated with constantly enhancing distributed intelligence of agents that is focussed on achieving better results as a group than any individual agent through individual enrichment and mutual recognition (Ha and Tang, 2022). Decentralization, diversity, and independence are all attributes that impact the potential of an agent system that possesses collective intelligence. As such, resilience is an invariable feature that should be enhanced with regard to maintaining autonomy and collective intelligence of systems. Resilience is not simply about having adaptive controls; rather, it refers to having the capacity to anticipate and respond to dynamic conditions. Therefore, the focus on resilience as discussed in this paper is a timely requirement to enhance the potential of autonomous agent systems.

Four primary attributes of resilience are identified here that characterize the ability to retain and/or adjust the functioning of the system in agent systems, namely: robustness; flexibility; scalability; and modularity. Robustness is the ability of a system either to preserve a certain property (such as the stability or performance) or to continue operations amid the presence of partial or individual failures, uncertainties, and disturbances to the environment (Gazi and Fidan, 2007). It does not cover the ability to anticipate and respond to adverse impacts. For agent systems with time delay, robustness has generally been implemented using approaches such as continuous time consensus protocols (Olfati-Saber and Murray, 2004) and other scenarios concerned with disturbances and model uncertainties have also looked at introducing foraging controllers to agents and sliding-mode control technique which reduces the motion of a system to a lower dimensional space (Gazi, 2005). Flexibility refers to the capacity to adapt to changing environments and requirements (Bayindir and Şahin, 2007). This should not be confused with robustness, as flexibility relates to identifying and shifting to suitable new behaviors to solve a different problem that was a consequence of the changed circumstances. Proactive agent control is gaining attention as an approach used to facilitate flexibility in an agent system where multiple agents, and potentially humans, collaborate to operate the tasks based on each others' capabilities, resources, and goals (Li et al., 2023). This enables flexible management of tasks in dynamic and uncertain circumstances. Agile scheduling using negotiation mechanisms (Rabelo et al., 1999) is another common approach used to improve the integration of information, coordination, and communication in agent systems targeting flexibility. The potential of the agent system to operate under a range of agent group sizes is known as scalability (Şahin, 2005). Decentralized and self-organized systems using approaches such as component distribution and replication (Deters, 2001), are capable of supporting the functioning of a group of agents irrespective of unexpected adversities such as malfunctioning agents, with a relatively low impact on performance. Lastly, modularity refers to the ability to use individual components, to operate on multiple and diverse functions and configurations to better pursue dynamic and complex environments (Beni, 2005). A modularized system can use individual agents interchangeably within the system for different tasks more easily than a single centralized system. Improving capability relations (Nunes, 2014) including association, composition, and generalization within agents is a common approach that is utilized in facilitating modularity of agent systems. Ideally, a resilient autonomous agent system should be able to adopt each of these respective attributes in response to the diverse environments and interactions it encounters.

The state-of-the-art in achieving resilience in agent systems have mostly focussed on decentralized control architectures (Ghedini et al., 2018; Sartoretti et al., 2019; Zhang et al., 2020; Blumenkamp et al., 2022). As discussed by Iocchi et al. (2001), a decentralized architecture could be distinguished from a centralized architecture based on how the decision-making process is designed. In a centralized system, there exists a central governing unit, an entity, or an agent that is in-charge of organizing and allocating tasks to the rest of the agents in the system. There are also weak centralized architectures where the central unit or the leader is dynamically assigned (Swaminathan et al., 2015) or the trajectory of the leader is dynamically decided (Rezaee et al., 2021) based on the environmental changes or interruptions and failures. In contrast, a decentralized control architecture (also referred to as distributed control architectures) has no central authority or a leader and the decision-making process is distributed among all agents in the system. These architectures have proven to be more scalable compared to centralized architectures as the computational burden of having a central controlling unit is mitigated increasing adaptivity in dynamic environments (Ismail and Sariff, 2018). However, they are not without limitations. Efficient task distribution is still challenging in adverse circumstances as the impacts cannot be anticipated beforehand (Steele and Thomas, 2007). Decentralized systems can reorganize the task allocation strategy but real-time adaptation can introduce unforeseen issues due to conflicts in capacity and skills associated with individual agents in the system.

Improving redundancy is another approach that has been used to achieve resilience in agent systems (Hazon and Kaminka, 2005). An agent system can be redundant in terms of the number of agents that can address a functionality or in terms of the number of approaches that a single task can be executed by the system. A failure or malfunction of a single agent or a functionality can be addressed by another agent or a different strategy in a redundant system. Therefore, it has been proven that redundancy can be leveraged to achieve parallelization in task execution while withstanding individual failures (Hsieh and Mather, 2013). However, coordinating and resolving the redundancies in a system is still a challenge and requires the exploitation of different managing criteria (Krizmancic et al., 2020). Further, these systems can often only vary within a defined spectrum of functions/parameters/sensory attributes and are not equipped with the decision-making capacity to identify new strategies or solutions to problems in real-time under dynamic circumstances.

Facilitating agent systems with distributed sensing capabilities such that multiple agents could sense the environment, can increase the signal-to-noise ratio of the system. This can help detect changes in the environment and identify and track anomalies and adversarial impacts on the system while collaboratively mapping dynamic environments (Bossens et al., 2022). Similar measures also include approaches such as distributed diffusion where information collected from different sensors or input sources of multiple agents is incorporated and the aggregation is used in decision-making of each agent in the system (Li et al., 2020). While distributed sensing facilitates robustness and increased relevancy of sensory measurements, the decision-making process after collecting this information has to be separately addressed.

The investigation of the said state-of-the-art approaches for achieving resilience demonstrates that these models suffer from limitations associated with anticipating dynamic and adverse environmental conditions. While they facilitate adaptive controls across a bounded range of potential dynamics, they lack the capacity to analyse an existing situation and make insights into unforeseen circumstances as a means of making predictions or preparations to adapt to these environments.

## 3. Counterfactual learning as a means to interpret machine learning models

In this section, the concept of counterfactuals is introduced as discussed within the literature on causal inference and how it has evolved as counterfactual learning which is an emerging technique used in explainable AI to improve the interpretability of machine learning models.

Counterfactuals have long been used in inferring causal relations (Lewis, 1974). They enable explaining past events and outcomes, and also predict future ones through links between causes and effects. They are essentially *what-if* questions; or conditional assertions where the antecedent is false, and the consequent describes what would have happened if it was true. Counterfactuals take a similar form to the following: “You were denied a loan because your annual income was $30,000. If your income had been $45,000, you would have been offered a loan” (Wachter et al., 2017). In this example, the statement of decision states the current representation of the world. The counterfactual that follows, describes what needs to change for the representation of the world to be favorable. As such, counterfactuals are, ideally, inferences that are made based on alternative possibilities.

Research in AI and machine learning has started recognizing the knowledge of counterfactuals as a useful attribute in multiple fields such as computer vision, natural language processing (NLP), and recommender systems. It is used as a means to supplement incomplete data and partial feedback received from learning models to improve performance through estimations and inferences. In computer vision models, counterfactual analysis has been used to clarify the existences of biases. For example, Joo and Kärkkäinen (2020) uses a model that generates counterfactural images across the dimensions of race and gender to evaluate the fairness of image classification techniques. Counterfactual image generation methods are also used in open set recognition (Neal et al., 2018) to train classifiers to detect unknown classes which are close to the training examples yet do not belong to any training category. Similar to computer vision models, NLP models have also utilized counterfactuals to mitigate annotation biases in datasets through causal inference. Tian et al. (2022) propose a counterfactual reasoning framework to mitigate the biases formulated in tasks of natural language inference, and Zhu et al. (2020) use counterfactual reasoning for open-domain dialogue generation where outcomes of alternatives for trained policies are used in the learning framework to expand the search space for higher performance levels. In recommender systems, counterfactual estimators enable investigating how a particular recommendation policy would have performed if it had been used instead of the policy that was used (Saito and Joachims, 2021).

Among these, the most prominent applications of counterfactuals in AI are perhaps seen in explainable AI (XAI) where it is commonly used as a model-agnostic and post-hoc technique to improve explainability in machine learning models. XAI is conceptualized in three phases of explanation which explore the aspects of: explanation generation, explanation communication, and explanation reception (Neerincx et al., 2018). In this regard, XAI is discussed across perceptual and cognitive categories. Perceptual XAI is concerned with providing perceptual foundations of agent behavior and the AI architecture used; whereas cognitive XAI is associated with describing why a certain action was chosen by relating them to the agents' beliefs and goals (Neerincx et al., 2018). Existing XAI approaches include the use of: saliency maps that describe the important aspects of inputs to a system (Adebayo et al., 2018); explainable BDI (belief-desire-intention) agent architecture that provides reasoning for changes in behavior (Harbers et al., 2012); and fuzzy-cognitive maps that can simulate the behavior of systems under different conditions to explain potential outcomes and decision making scenarios (Apostolopoulos and Groumpos, 2023), among others. Causal inferences generated through counterfactual learning could be used in two aspects: local and non-local in generating XAI models for agent systems in contrast to the other existing approaches. Local counterfactual learning focuses on what features, attributes, or characteristics would need to be changed to achieve a certain outcome (Chou et al., 2022). Further, non-local counterfactuals can be used to determine absence causations which identify the lacking, failure, or non-occurrence of attributes or characteristics that lead to certain outcomes of the system (Dowe, 2009). The dynamic and ad-hoc explanations of these models and the understanding of their decision-making process can be utilized to improve the accuracy of the models as well as in increasing the trust of human users in these systems (Byrne, 2019).

## 4. Proposed counterfactual learning-based approach for resilience in autonomous agent systems

The existing AI applications of counterfactual learning discussed in Section 3 have identified the means of using the causal relations generated by counterfactuals to further analyse and gain insights into these applications. Based on this understanding, this paper proposes that the exploitation of the causal inferences that can be extracted through counterfactuals is useful in enhancing the resilience of autonomous agent systems in terms of analysing the present experiences to foresee and prepare for dynamic environmental conditions that are beyond the boundaries of experienced outcomes.

**Figure 1:**
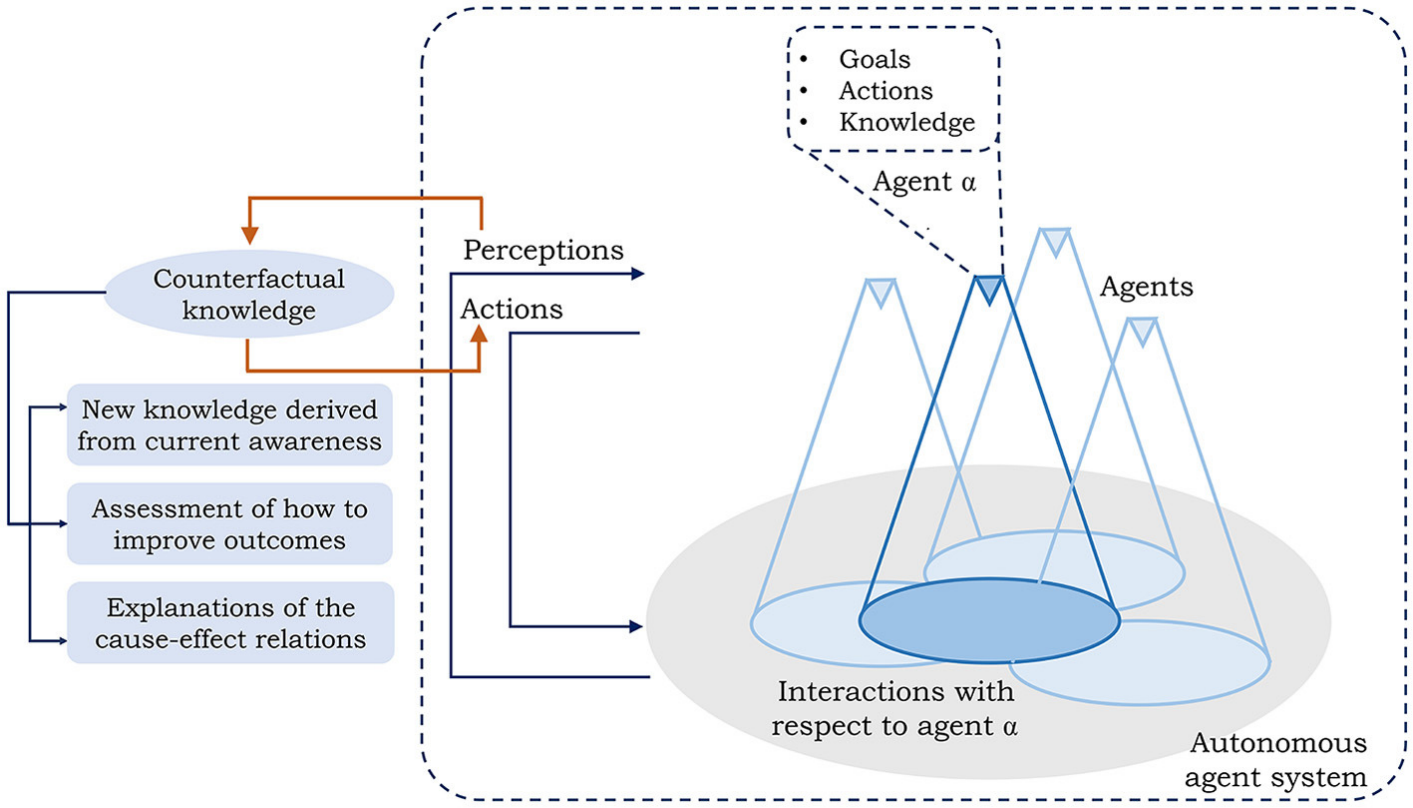
Incorporating counterfactual knowledge in an agent system within an active learning environment. Each agent is endowed with prior domain knowledge, actions, and goals which are used in building the perception of the current world. The counterfactual knowledge derived from the current perceptions can influence the actions and thus the performance of the system.

Figure 1 elaborates how the knowledge of counterfactuals can be integrated in an agent system. In a typical agent-based environment, the agent(s) are endowed with pre-knowledge on the domain, a set of actions, and goals. As each agent senses and interacts with the other agents and/or the environment, they build perceptions which result in actions that affect the neighboring environment (Samarasinghe et al., 2022b). The said perceptions can be used in generating the counterfactual knowledge in an active learning environment to influence and improve the actions and thus the performance of the system. Three primary avenues on how counterfactuals can facilitate resilience in agent systems are focussed in this aspect. In this regard, this paper identifies that counterfactuals can be used to:

- Generate new knowledge for agents based on their current awareness of the system.
- Describe and assess the decision-making process of agents in the system.
- Construct explanations of cause-effect or reason-action relationships between events and agents in the system to improve the implementation architecture.

As the first approach, counterfactuals can be used to generate new knowledge based on the current awareness on a system, its operations, and the environment it is operating in. They have been used in multiple applications for agent systems to supplement incomplete and/or limited data. Counterfactuals can be used to analyse the current situation and envisage the different outcomes by changing the antecedents to add to the knowledge. Counterfactual predictions are used by Jin et al. (2022) to guide the exploration of a reinforcement learner for robotic manipulation tasks. The active counterfactual predictions generated add to the existing body of knowledge about the task for the learning model to improve its performance. Mannion et al. (2016) propose a novel reward shaping technique to improve multi-agent reinforcement learning (MARL) for autonomous control of agents in complex environments. The method they discuss: Estimated Counterfactual as Potential, is capable of generating new potential functions to shape the behaviors of the agents, using approximations of knowledge on different evaluations using counterfactuals. The estimated potentials of the agents can help these agents outperform the agents learning only with system evaluation functions. Further, counterfactual multi-agent policy gradients are also used with similar intentions to estimate new knowledge to optimize agent policies learned with machine learning models (Foerster et al., 2018). However, the expected new knowledge could be unique and belong to a different dimension from the current knowledge due to changed environmental conditions and interactions. Therefore, it should be noted that approaches such as logical autoregression and extrapolation which impose strong assumptions on the agents' rationality and the dynamics of the environment may not always be accurate. Methods such as Robust Multi-Agent Counterfactual Prediction (RMAC; Peysakhovich et al., 2019) can be utilized to analyse the sensitivity of counterfactual predictions and derived knowledge to violation of such assumptions.

Secondly, counterfactuals could be used as a means to describe or assess how the outcome of a certain decision-making process could have been better. Counterfactual regret minimization (CFR) is a widely adopted algorithm in solving large imperfect-information games (Brown et al., 2019). These games typically simulate real world domains including cybersecurity interactions, auctions, and negotiations which consist of partial information-based strategic interactions between multiple agents. CFR algorithms can train agents to solve problems under such conditions through self-play. The current strategy of each agent is improved by comparing it to others through calculating a *counterfactual regret* for not taking an action at a particular state. This regret represents a quantitative approximation of how good the outcome would have been had it picked a certain action based on the information it gains by observing the system. Counterfactual reasoning is also used in interactive environments for motion planning through inferences about other interacting agents that could have led to improved results/performance. Inference about potential motion of other agents derived through counterfactuals is used by Bordallo et al. (2015) to understand how the current motion plan could be improved in a real-time learning setting. Efficient iterative planning is achieved for a multi-robot navigation scenario through the predictions made on how good the outcome could be, by observing the actions of other agents.

Finally, the construction of explanations through the identification of cause-effect or reason-action relationships between events can further enhance the resilience of agent systems by incorporating the insights thus gained into the implementation architecture. They can enable users and designers of such systems to make inferences such as Modus Tollens (of the form: *If P, then Q. Not Q. Therefore, not P*) through simulation of multiple possibilities of interactions and events which is otherwise strenuous within the limits of human cognitive capacity. Mueller et al. (2021) propose a visual interactive model for robot skill learning from visual demonstrations. An interactive augmented reality-based approach is used to iteratively program robot skills through the demonstrations. They utilize Robot Behavior Counterfactuals (RBCs) to provide *what-if* visualizations to explain the effects that alternatives and constraints have on adapting skills to altered environments. The behavior verification indicators provided by the counterfactuals support the users in gaining a better understanding of the model and when and where it can be applied and succeed or fail to further improve the robot training models. Similarly, counterfactual explanations have also been used in envisioning the potential movement paths in autonomous robotic control while coping with the disturbances and uncertainties in the environment. In Smith and Ramamoorthy (2020), a generative model to produce counterfactual disturbances is proposed as a means to characterize the potential of the robot controls. They are used to explain the perception-action relations which in turn, can be used to modify the configurations to achieve an expected outcome.

## 5. Conclusion

Intelligent agent systems demand the need for automation that can facilitate active learning, adaptation, and evolution in order to be able to sustain in complex and dynamic environmental niches without direct human supervision. Therefore, such systems should be resilient, i.e., should have the capacity to anticipate, respond to, adapt to, and recover from adverse and dynamic conditions in complex environments. In particular, resilient agent systems should be able to generate agile policies that can predict and overcome adverse conditions that are beyond the modeled boundaries (Prorok et al., 2021). As such, a resilient system should possess the four attributes: robustness, flexibility, scalability, and modularity. As discussed in Section 2, each of these attributes uniquely characterize different aspects of resilience. Robustness is the ability to preserve the functionality of the system in partial failures, whereas flexibility is associated with the ability to adapt to suitable new behaviors when the environment and circumstances change. Scalability relates to the potential of the agents to operate with different agent group sizes facilitating failed or newly added agents in the system. Modularity covers the ability to use individual components/agents of the system to operate different functionalities. A resilient agent system should encompass all these aspects to be successful in interacting with dynamic environments. In this perspective, this paper proposes the use of counterfactual knowledge as a means to facilitate resilience in agent systems with its ability to make inferences about existing causal relations and predictions on future impacts.

The avenues discussed in Section 3 have provided evidence that they can enhance the boundaries of the current agent learning models that are limited in their capacity to anticipate and react to unmodeled unforeseen scenarios. In order to cater to all four attributes of resilience, an ensemble of all three avenues of counterfactuals: generating new knowledge to understand the potential of the agent system; assessing how the outcomes of decision-making processes could improve, and explaining the cause-effect relationships between the events; is suggested as the way forward. The implementation of the model could take an iterative evolutionary approach where the counterfactual knowledge generated from the three avenues influence the actions of the agent system which in turn feed the evolutionary model as the current perceptions to generate new counterfactual knowledge in the subsequent iteration. The generation of new knowledge based on the current awareness can be made possible with the use of an Estimate Potential approach for counterfactuals as discussed in Section 4. The explanations of the cause-effect relationships of the current system can be derived from these counterfactuals that can enhance the understanding of the decision-making process of the agents. With the explanations, the new potential functions generated can be used to shape the agent behaviors in conjunction with the existing knowledge. An RMAC method can be utilized to analyse the sensitivity of the counterfactual knowledge to the dynamics of the environment, which can inform the decision to of selecting the most robust actions. CFR approach discussed in Section 4 can be used as a mean to assess whether and how the current decision process of the system could be improved based on comparisons against the derived counterfactuals. The combined knowledge derived from the union of these counterfactual-based approaches can then be used to identify the most suitable actions for the agent system. The variations in the environment and consequences of the actions can then be reiterated as perceptions to feed the counterfactual knowledge module to generate the next set of actions for the system.

The said model can enhance the four aspects of resilience (robustness, flexibility, scalability, and modularity) facilitating intelligent autonomous agents that can better perform in dynamic and complex environments. As expected of a robust system, the ability to preserve functionalities and continue operation amid partial failures can be ensured through the understanding of causal inferences of the current domain provided by counterfactual analysis. As discussed by Peysakhovich et al. (2019), counterfactuals can be used to achieve robust performance across different environments with changing rules. The flexibility to switch to new behaviors as a consequence of changed circumstances and requirements is ensured through the avenues that explore counterfactuals in generating new knowledge based on the current perceptions. Both scalable and modular characteristics of an agent system that can operate under a range of agent group sizes and optimally utilize different components (agents or skills of individual agents) of the system to operate multiple functions, can be facilitated through the knowledge of current cause-effect relationships and the understanding of how the outcomes of the decision-making process can be improved. This can enable appropriate distribution of tasks among the available number of agents based on their individual skills and dynamic requirements that arise (Singh et al., 2021).

As identified, counterfactual learning has the potential to advance the future of intelligent resilient agent systems. However, it is not without limitations. Specifically in agent system, there exists a requirement for the counterfactual explanations and new knowledge generated to represent the system's operation along causal, justificatory, and purposive lines (Arnold et al., 2021). As counterfactuals can make inferences and predictions resulting in actions that are outside the boundaries of the modeled circumstances, another layer of validation could be required based on determinants and constraints on the course of actions the agents can take. Further, there exist a tendency for counterfactuals to be unstable with respect to the changes in the environment (Artelt et al., 2021). A small change in the input can result in a drastic change in the output. As a result, different actions recommended may have arbitrary changes in complexity which might cause issues in practicability of application. Therefore, incorporation of counterfactuals require careful consideration and further research into how the limitations can be overcome. However, the discussion presented in this paper lends valuable insights into the potential of counterfactuals as a successful alternative to model resilient autonomous agent systems to address the complex and dynamic requirements of the real-world applications.

## Data availability statement

The original contributions presented in the study are included in the article, further inquiries can be directed to the corresponding author.

## Author contributions

DS conceived the idea of the paper, prepared, and reviewed the article.
